# Mutation Hotspot for Changing the Substrate Specificity of β-*N*-Acetylhexosaminidase: A Library of GlcNAcases

**DOI:** 10.3390/ijms232012456

**Published:** 2022-10-18

**Authors:** Pavlína Nekvasilová, Natalia Kulik, Michael Kotik, Lucie Petrásková, Kristýna Slámová, Vladimír Křen, Pavla Bojarová

**Affiliations:** 1 Laboratory of Biotransformation, Institute of Microbiology of the Czech Academy of Sciences, Vídeňská 1083, CZ-14220 Praha 4, Czech Republic; 2 Department of Genetics and Microbiology, Faculty of Science, Charles University, Viničná 5, CZ-12843 Praha 2, Czech Republic; 3 Laboratory of Structural Biology and Bioinformatics, Institute of Microbiology of the Czech Academy of Sciences, Zámek 136, CZ-37333 Nové Hrady, Czech Republic

**Keywords:** β-*N*-acetylhexosaminidase, *Talaromyces flavus*, *Pichia pastoris*, site-saturation mutagenesis, site-directed mutagenesis, substrate specificity

## Abstract

β-*N*-Acetylhexosaminidase from *Talaromyces flavus* (*Tf*Hex; EC 3.2.1.52) is an *exo*-glycosidase with dual activity for cleaving *N*-acetylglucosamine (GlcNAc) and *N*-acetylgalactosamine (GalNAc) units from carbohydrates. By targeting a mutation hotspot of the active site residue Glu332, we prepared a library of ten mutant variants with their substrate specificity significantly shifted towards GlcNAcase activity. Suitable mutations were identified by in silico methods. We optimized a microtiter plate screening method in the yeast *Pichia pastoris* expression system, which is required for the correct folding of tetrameric fungal β-*N*-acetylhexosaminidases. While the wild-type *Tf*Hex is promiscuous with its GalNAcase/GlcNAcase activity ratio of 1.2, the best single mutant variant Glu332His featured an 8-fold increase in selectivity toward GlcNAc compared with the wild-type. Several prepared variants, in particular Glu332Thr *Tf*Hex, had significantly stronger transglycosylation capabilities than the wild-type, affording longer chitooligomers – they behaved like transglycosidases. This study demonstrates the potential of mutagenesis to alter the substrate specificity of glycosidases.

## 1. Introduction

β-*N*-Acetylhexosaminidases (EC 3.2.1.52, CAZy GH20; http://www.cazy.org (accessed on 5 July 2022)) are naturally hydrolytic enzymes that catalyze the cleavage of *N*-acetyl-β-d-glucosaminyl (GlcNAc) and *N*-acetyl-β-d-galactosaminyl (GalNAc) residues at the non-reducing ends of oligosaccharide chains [1,2]. In vitro, the glycosidase-catalyzed reaction may be shifted in favor of transglycosylation by modifying the reaction conditions (e.g., by using activated glycosyl donors or optimizing the donor/acceptor ratio) or by medium engineering, which besides the synthetic yield, also enables to steer substrate specificity by the addition of salts [3] or organic solvents [4]. Recently, genetic engineering of β-*N*-acetylhexosaminidases has brought very promising results [5]. Site-directed mutagenesis [6], domain-targeted mutagenesis [7], or loop-engineering [8] have been applied to improve the transglycosylation abilities of β-*N*-acetylhexosaminidases [5].

In general, the specificity of wild-type (WT) fungal β-*N*-acetylhexosaminidases varies between species. There are relatively selective GlcNAcases: β-*N*-acetylhexosaminidase from *Aspergillus oryzae* (*Ao*Hex) with a GalNAcase/GlcNAcase ratio of 0.3–0.6 [9], or from *A. versicolor* (*Av*Hex) with a GalNAcase/GlcNAcase ratio of 0.09–0.11 [10]. There are also prevalent GalNAcases, e.g., β-*N*-acetylhexosaminidase from *Penicillium oxalicum* (*Po*Hex) with a GalNAcase/GlcNAcase ratio of 1.4–2.3 [9,11]. Although bacterial β-*N*-acetylhexosaminidases capable of GalNAc transfer have been identified [12,13,14], selective β-GalNAcases are quite rare [9,11]. They have so far been described in only a few bacterial species, namely *Bacteroides* [15], *Clostridium* [16], and *Paenibacillus* [17], but they have not been shown to possess transglycosylation abilities. Pure *O*-GlcNAcases can be found in the family GH84 [18], which do not exhibit any transglycosylation abilities and contain only minor traces of GalNAcase activity [19]. Importantly, many alleged GlcNAcases also harbor a substantial GalNAcase activity, such as *Bifidobacterium bifidum* β-*N*-acetylglucosaminidase with a GalNAcase/GlcNAcase ratio of 1.4, because in many cases, the GalNAcase activity is simply not determined. However, for discrimination in carbohydrate mixtures or for selective syntheses, a high level of selectivity between both β-*N*-acetylhexosaminidase activities is required.

β-*N*-acetylhexosaminidase from *Talaromyces flavus* (*Tf*Hex) is well known for its excellent transglycosylation ability and broad substrate specificity [20,21,22]. The WT enzyme, a typical promiscuous β-*N*-acetylhexosaminidase, is characterized by a GalNAcase/GlcNAcase activity ratio of 1.2 [23]. Interestingly, though the catalytic domain of *Tf*Hex shares quite a high homology with *Ao*Hex (67% sequence identity) [24] and *Po*Hex (66% sequence identity) [25], the differences in substrate specificities of these enzymes are distinct. The specificity of β-*N*-acetylhexosaminidases is mainly determined by their primary amino-acid sequence, but there are also other factors that have not been fully elucidated, apparently related to, e.g., post-translational modifications and folding [23].

*Tf*Hex, even as a WT, has the quite unique property of tolerating various functions in the structure of its substrates. This enables to elegantly introduce functional moieties in an oligosaccharide, a feature widely applicable in the synthesis of labeled or multivalent glycostructures for biomedical research [4,21,26]. Its mutagenesis thus expands the library of enzyme tools available for preparative biotransformations. Several engineering strategies have been employed to alter the catalytic properties of fungal β-*N*-acetylhexosaminidases. The pioneering approach to suppress the hydrolytic activity by substituting the residue Tyr470, the stabilizer of the substrate *N*-acetyl group [27], resulted in a significant increase in the transglycosylation activity of *Tf*Hex. The substitution of the residue Asp370 stabilizing the substrate *N*-acetyl group during transition state formation [28] afforded glycosynthase-like variants with retained transglycosylation activity and minor hydrolytic activity that did not process α-GlcNAc fluoride as a glycosyl donor [29,30] but only the standard *p*NP-β-GlcNAc. The combined mutation of the active site tyrosine and aglycone-binding residues generated superior hypertransglycosylating variants of *Ao*Hex [31]. Another approach to modifying *Tf*Hex substrate specificity was the substitution of the conserved residue Glu546 in the −1 subsite, resulting in a quite selective GalNAcase used for the synthesis of a GalNAc-terminated trisaccharide with a (GlcNAc)_2_ core [32]. Unselective β-*N*-acetylhexosaminidases would not be able to accomplish such a synthesis because they would readily cleave the acceptor. *N*-Acetylhexosamine oligosaccharides are widely applicable for targeting lectins, such as wheat germ agglutinin [33] or galectins [34,35]. The use of tailored glycosidases may thus be a good alternative to the use of glycosyltransferases [36].

**Figure 1:**
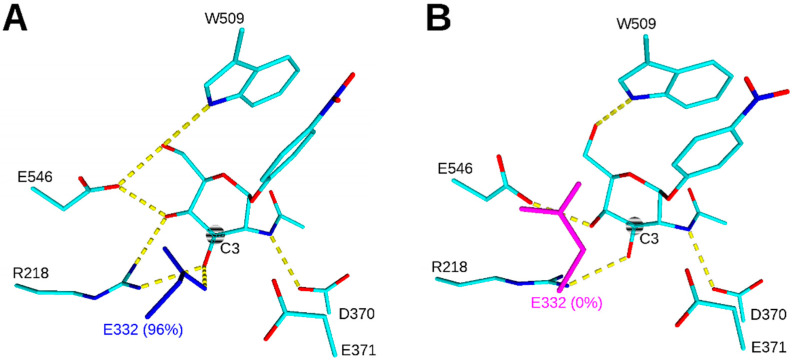
Position of substrates in the active site of *Tf*Hex WT after 20 ns of molecular dynamics simulation. (**A**) *p*NP-GlcNAc with Glu332 in blue; (**B**) *p*NP-GalNAc with Glu332 in magenta. The frequency of occurrence of a hydrogen bond between the substrate C-3 hydroxyl and Glu332 during 15–20 ns of molecular dynamics simulation is shown in brackets. Hydrogen bonds are shown in yellow dashed lines, and hydrogen atoms are hidden. The substrate C-3 atom is highlighted and labeled.

In the present work, we have thoroughly investigated the relationship between a point mutation introduced into the +1 subsite of the active site and its effect on the activity and substrate specificity of *Tf*Hex. According to the homology modeling of *Tf*Hex WT (Figure 1) [37,38], Glu332 was identified as one of the crucial amino acids close to the substrate C-4, involved in the direct interaction with only GlcNAc substrate (C-3 atom). This residue is not highly conserved among GH20 β-*N*-acetylhexosaminidases, e.g., it is substituted by Gln in *Av*Hex, which has a high preference for GlcNAc substrate [10]. Therefore, this residue was selected as the mutation hotspot to alter the *Tf*Hex substrate specificity. In the first approach, we applied site-saturation mutagenesis, which, however, yielded only limited results in our hands. Therefore, we resorted to site-directed mutagenesis, where the prospective variants were proposed by in silico modeling. Thus, we could thoroughly elucidate the relationship between the amino acid substitution in the active site and the change in the enzyme properties. We found that the point mutation at this position prevalently led to an increase in GlcNAcase activity relative to GalNAcase. In the series of ten variants prepared, we identified Glu332His *Tf*Hex, which had a GalNAcase/GlcNAcase ratio (0.15) comparable to the most selective natural fungal β-*N*-acetylhexosaminidase from *A. versicolor* [10].

## 2. Results

Our previous docking and molecular dynamics simulation study of interactions between the active site residues of *Tf*Hex and its substrates revealed that the side-chain of Glu332 is located close to the C-3 and C-4 hydroxyls of the substrate and could form hydrogen bond interaction with *p*NP-GlcNAc while the hydrogen bond with *p*NP-GalNAc was not formed, which often affects the binding affinity to the substrate (Figure 1) [32]. Thus, due to the direct involvement in the interaction with the substrate, the mutation of the Glu332 residue was proposed to significantly shift the substrate specificity either sterically, energetically, or both. Therefore, this residue was selected as the mutagenesis hotspot for the present study.

### 2.1. Site-Saturation Mutagenesis Approach—A Dead-End Street?

In the first approach, we decided to perform site-saturation mutagenesis of the *Tf*Hex gene at the position of Glu332 (Supplementary Table S1). Due to their complex quarternary structure, β-*N*-acetylhexosaminidases need to be produced in a eukaryotic host such as *Pichia pastoris*. Advantageously, *P. pastoris* is capable of complex post-translational modifications, and in particular, the correct folding of the catalytically active tetramer consisting of two non-covalently associated subunits and two non-covalently linked propeptides [39], and glycosylation, contrary to the prokaryotic host of *E. coli* [40,41]. The WT gene of *Tf*Hex was cloned into the pPICZαA expression vector using *Eco*RI and *Kpn*I restriction sites downstream of the α-factor (for extracellular enzyme targeting and zeocin resistance) as described previously [39]. The site-saturation mutagenesis was performed by PCR as detailed in Materials and Methods. After the transformation of competent cells of *P. pastoris* KM71H, we initially screened 150 clones as recommended by Reetz et al. [42,43] (see Equation (1) in Section 4-Materials and Methods) to cover all 19 amino acid residues except for the WT (Supplementary Table S1). However, because the activity screening afforded mostly variants with a GalNAcase/GlcNAcase ratio close to the WT (ca. 0.6–1.4), we increased the number of screened colonies to approximately 1600. For this aim, we optimized the screening of transformed *P. pastoris* cells in 96-deep-well plates under methanol induction based on the procedure by Weis et al. [44]. The deep-well screening was performed in the minimal medium with glucose as a carbon source, with 6-day-long cultivation. The production of active enzymes (GalNAcase & GlcNAcase activities) in the cultivation medium was confirmed by the spectrophotometric activity assay using *p*NP-GalNAc and *p*NP-GlcNAc substrates.

The 22 prospective variants with a significantly modified GalNAcase/GlcNAcase ratio (more than 3-fold decreased or increased compared to the WT) were then produced by the standard cultivation procedure in flasks and purified by cation-exchange chromatography. Thus, we could confirm or disprove the preliminary estimate of the activity ratio from the medium screening. As a result, only seven variants were selected for further analysis, in which the significant shift in activity ratio was confirmed after purification. The respective Glu332 mutation in the mutant variants was identified by DNA sequencing. Chromosomal DNA of *P. pastoris* was isolated by combining enzymatic degradation (zymolyase) and adsorption (DNeasy Ultraclean Microbial Kit) to reach sufficient amounts of DNA for further processing. After amplification of mutant *Tf*Hex genes by PCR, they were ligated into the pGEM-T Easy vector and sequenced.

The properties of the analyzed clones selected for DNA isolation are summarized in Supplementary Table S2. In summary, as a result of site-saturation mutagenesis, we identified Glu332Gly, Glu332His, and Glu332Trp *Tf*Hex variants with a significantly shifted GalNAcase/GlcNAcase activity ratio (Supplementary Table S2). In one case, we detected simultaneous recombination of two different genes into the yeast genome in the variant Glu332Trp/Glu332Arg. Although multiple gene copies are usually desirable due to higher expression levels of recombinant proteins [45,46], and may further reinforce the desired enzyme property, multiple gene expression hinders a straightforward elucidation of structure-activity relations. In our case, the combined Trp/Arg variant showed a quite favorable GalNAcase/GlcNAcase activity ratio, but since it was not the best of all variants, we have not analyzed it further.

Despite the risk of multicopy expression, *P. pastoris* has previously been used for screening of directed-evolution mutants or products of DNA shuffling. Chen and coworkers identified two mutant α-galactosidases from *Penicillium janczewskii* zalesk with a higher galactosidase activity, potentially useful in feed manufacturing [47]. Kao and coworkers used a 96-deep-well plate screening to identify a β-glucosidase with an enhanced specific activity [48]. However, since this system afforded just limited results for the site-saturation mutagenesis of *Tf*Hex (only four prospective amino acid exchanges were identified), we resorted to the site-directed mutagenesis approach, as detailed further.

### 2.2. Site-Directed Mutagenesis Approach

For the aim of site-directed mutagenesis, we had to predict the potential candidate mutations at Glu332 by molecular modeling.

#### 2.2.1. In Silico Rational Design of Prospective *Tf*Hex Mutants

**Figure 2:**
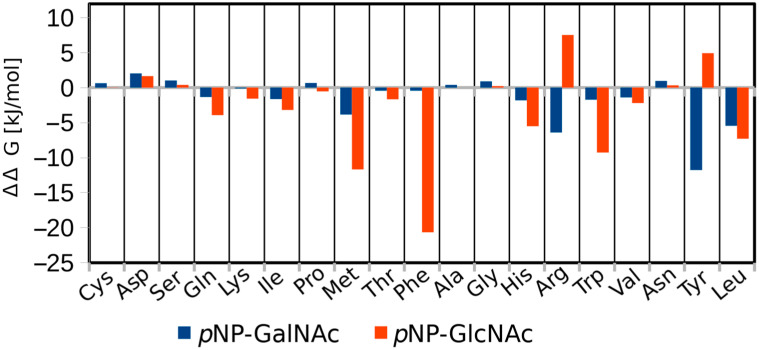
Change in the free energy of binding (ΔΔ*G*) of the enzyme-substrate complex compared to the WT enzyme (ΔΔ*G*_WT_ = 0 kJ/mol). Change in GlcNAcase activity (affinity to *p*NP-GlcNAc) is shown in orange, and change in GalNAcase activity (affinity to *p*NP-GalNAc) is in blue.

The in silico modeling of interactions between β-*N*-acetylhexosaminidases and their substrates is a relatively challenging task, given the high substrate flexibility of the enzyme. Additionally, the predictions acquired for *Tf*Hex are just based on the homology model with the enzyme from *Ao*Hex (sequence identity of catalytic domains is 67%), which is the only known fungal β-*N*-acetylhexosaminidase with a resolved 3D structure [38]. These limitations had to be considered when analyzing the results of in silico experiments. The final selection of potential candidate mutant variants of *Tf*Hex with a shifted GalNAcase/GlcNAcase ratio was performed based on a combination of in silico methods. One of them was the scanning of residues by BioLuminate based on MM-GBSA scoring (a part of Schrödinger software, release 2018-4) [49]. By using this program, the Glu332 residue was in silico mutated into the corresponding amino acid residue, followed by short energy minimization in an implicit solvent (including only residues within 5 Å of the mutated residue). Then, the change in the substrate-enzyme binding affinity compared with WT based on MM-GBSA scoring (ΔΔ*G* = Δ*G*_mutant_ − Δ*G*_WT_) was calculated. More negative values of ΔΔG corresponded to variants with an enhanced preference for the respective substrate over WT. The discrimination between *p*NP-GlcNAc and *p*NP-GalNAc should be achieved by selecting variants with a higher absolute difference in respective ΔΔG values (Figure 2). According to the ΔΔ*G* calculation, the exchange of Glu332 for Gln, Lys, Met, Thr, Phe, His, Trp, and Leu was predicted to improve the affinity to both substrates (*p*NP-GlcNAc and *p*NP-GalNAc) compared with WT, with a more pronounced GlcNAcase activity (Figure 2). In addition, two variants were expected to show improvement in GalNAcase activity compared to WT: Glu332Arg and Glu332Tyr *Tf*Hex.

Since the BioLuminate method only analyzes the interactions of a static complex, the hypothetical Glu332 mutant variants in the complex with both substrates were next subjected to molecular dynamics simulation. Thus, we could verify the stability of the formed complexes and analyze the interactions of the respective variant with the substrates. The molecular dynamics simulations of enzyme-substrate complexes were run in YASARA for 30–50 ns. The root-mean-square deviation (RMSD) analysis revealed that *p*NP-GlcNAc was not stabilized in the active site of Glu332Met and Glu332Arg *Tf*Hex and that neither of the substrates was well stabilized in the active site of Glu332Leu *Tf*Hex (Supplementary Figures S1 and S2). Therefore, these three variants were excluded from the BioLuminate selection. The complexes of variants with significant differences in substrate orientation required for hydrolysis were analyzed by visual inspection. All modeled complexes are shown in Supplementary Figures S2 and S3. The formation of hydrogen bonds, reflecting on the correct formation of the oxazolinium ion intermediate, is shown in Figure S4. For substrates docked in the selected candidate mutants, this parameter distance was comparable to WT.

The equilibrated complexes of both substrates with the variants allowed us to analyze the binding score for each substrate-enzyme complex for selected snapshots derived from the stable period of molecular dynamics simulation (Supplementary Table S5). The variants with a larger XP score difference between *p*NP-GlcNAc or *p*NP-GalNAc than WT should allow for better discrimination between both substrates than WT. They are denoted in bold in Table S5 (Phe, Gly, His, Asn). Based on this particular analysis, we can consider them as potential candidates for modulating the GalNAcase/GlcNAcase ratio. This extended the original BioLuminate-method list of prospective variants with Glu332Gly and Glu332Asn *Tf*Hex.

To complete the selection of candidate variants, we also included published mutant homologs in the analysis. For β-*N*-acetylhexosaminidase from *Ostrinia furnacalis*, the exchange of the residue corresponding to Glu332 for Ala and Gln was reported [50]. To compare the effects of these mutations on the substrate affinity in *Tf*Hex, we also included the Ala variant in the selection.

In summary, by combining (*i*) the results of the BioLuminate residue scanning showing altered free energy of binding; (*ii*) the analysis of the stability of the enzyme-substrate complex in molecular dynamics simulation; (*iii*) the binding scores for individual substrates in equilibrated complexes (Glide XP method); and (*iv*) information from the literature, a library of ten prospective variants of *Tf*Hex at the Glu332 hotspot (Ala, Asn, Gln, Gly, His, Lys, Phe, Thr, Trp, Tyr) was selected. These variants were prepared by site-directed mutagenesis and tested for their GalNAcase/GlcNAcase ratio.

#### 2.2.2. Preparation of *Tf*Hex Mutants by Site-Directed Mutagenesis

Based on molecular modeling, ten prospective amino acid substitutions at the position of Glu332 were selected for modifying *Tf*Hex substrate specificity. Single mutant variants were prepared by PCR from the WT gene [39], as summarized in Supplementary Table S3. The codons present in the respective variants are shown in Table S4. Some mutant constructs were also isolated while probing the occurrence of mutant genes during site-saturation mutagenesis (cf. Supplementary Tables S1 and S4). Mutant pPICZαA-*Tf*Hex plasmids were linearized by restriction endonuclease *Sac*I and electroporated into the competent cells of *P. pastoris* KM71H. After screening for growth under the selection pressure of zeocin, the production of variants was monitored by SDS-PAGE, and enzyme activity was determined with a standard activity assay. The clones exhibiting the most extreme ratio of GalNAcase/GlcNAcase activities were cryopreserved and aliquots stored at −80 °C.

#### 2.2.3. Production and Characterization of Site-Directed Mutants of *Tf*Hex

The preparative production of *Tf*Hex variants was performed in minimal cultivation medium for easier purification. The variants of *Tf*Hex were purified to homogeneity in a single purification step by cation-exchange chromatography. Their purity was confirmed by SDS-PAGE (Supplementary Figure S5). The purification yield and the specific GalNAcase and GlcNAcase activities were determined; their ratio was evaluated. The obtained data were compared with the recombinant *Tf*Hex WT [23] and are summarized in Table 1. The specific activities show that the mutagenesis generally caused a significant decrease in the GalNAcase/GlcNAcase activity ratio in the tested variants, affording GlcNAcases (3- to 8-fold more selective compared to WT). The Glu332Trp and Glu332Phe *Tf*Hex variants were rather unselective. The only variant that showed a (slightly) higher GalNAcase affinity than WT was Glu332Tyr *Tf*Hex with a respective ratio of 1.4.

Notably, in many prepared variants, the significant increase in selectivity (GalNAcase/GlcNAcase activity ratio decreased from 3- to 8-fold compared to WT) has not caused a considerable decline in specific activity as is otherwise usual in active site mutant variants, particularly those at the catalytic nucleophile [51]. Here, in many cases, we rather observed an increase in the enzyme specific activity compared with the WT. This was particularly striking for the Glu332Thr variant. Its specific (GlcNAcase) activity increased almost 4-fold compared with the WT. A higher specific GlcNAcase activity over WT was also observed in Ala, Asn, Gln, and Gly variants. The highest substrate selectivity was achieved by introducing histidine and lysine; however, in both cases, it was accompanied by a sharp decline in the specific activity. In the most selective variant Glu332His *Tf*Hex, the GalNAcase/GlcNAcase activity ratio decreased ca. 8-fold (ratio of 0.15), and in the lysine variant, ca. 7-fold (ratio 0.18) compared with the WT. The previously published Arg218Lys *Tf*Hex exhibited a GalNAcase/GlcNAcase ratio of 0.15 [32].

#### 2.2.4. Kinetic Analysis and Transglycosylation Potential of the Best Mutants

To gather more information on the influence of the Glu332 mutation on the catalytic activity, we determined the kinetic parameters of outstanding GlcNAcase variants. In all the variants with increased specific GlcNAcase activity relative to WT (Ala, Asn, Gln, Gly, and Thr; Table 1), we observed a well-preserved value of the turnover number (*k*_cat_) but a significantly higher *K*_M_ (i.e., lower affinity to the *p*NP-GlcNAc substrate). As a result, the catalytic efficiency (*k*_cat_/*K*_M_) in these variants decreased from 12- to 27-fold compared to WT *Tf*Hex [23], though it was still well comparable with other native GlcNAcases such as *Av*Hex (Supplementary Table S6).

In the two most selective variants (His and Lys), this trend was even more pronounced. Due to a very high *K*_M_ (>7 mM), the kinetic curves could not be measured to full saturation due to the limited water solubility of the *p*NP-GlcNAc substrate (Supplementary Figure S6). Therefore, for Glu332Lys *Tf*Hex, only estimates of kinetic parameters are presented [23]. Interestingly, Glu332Gln *Tf*Hex showed a unique catalytic behavior, which could not be fitted to the standard Michaelis-Menten kinetics (Supplementary Figure S6), and thus disabled a reliable extraction of kinetic parameters for this variant. We suspect that this phenomenon may be caused by changes in the hydrogen bonding network during substrate binding in the active site caused by the Gln332 substitution.

**Figure 3:**
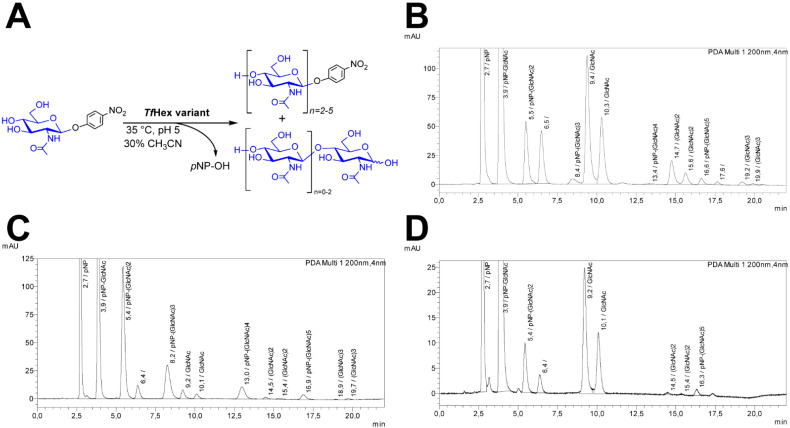
(**A**) Enzymatic synthesis of functionalized chitooligomers catalyzed by *Tf*Hex variants. (**B**–**D**) Comparison of formation of *p*NP-chitooligomers under the catalysis by (**B**) *Tf*Hex WT [32], (**C**) transglycosylating variant Glu332Gly *Tf*Hex, or (**D**) hydrolyzing variant Glu332Lys *Tf*Hex, using *p*NP-GlcNAc both as a donor and an acceptor in the same starting concentration and the same concentration of enzyme (U/mL) for all reactions. The reaction was analyzed after 5 h. The analysis was performed on a TSK gel Amide-80 column (for details see Section 4). For reducing carbohydrates, two peaks are visible due to the separation of α- and β-anomers.

The transglycosylation ability of *Tf*Hex variants was tested in a self-condensation reaction with *p*NP-GlcNAc. This experiment showed the effect of the introduced mutation on the transglycosylation capability of the enzyme. Previously, the formation of longer *p*NP-functionalized chitooligomers was described in the reaction with *Tf*Hex transglycosidases [35] and with Arg218Lys *Tf*Hex [32]. We used the same reaction conditions as in our previous work [32] (100 mM *p*NP-GlcNAc; 1 U/mL enzyme) for the analytical reaction with outstanding *Tf*Hex variants and compared it with the respective data for *Tf*Hex WT (Figure 3). Interestingly, in contrast to WT and also to the previously described Arg218Lys mutant variant with a comparable GalNAcase/GlcNAcase activity ratio, we found that Glu332His and Glu332Lys *Tf*Hex had a completely different transglycosylation behavior. Instead of creating a whole array of transglycosylation products (chitooligomers of varying lengths), these variants selectively formed only a small amount of *p*NP-(GlcNAc)_2_, behaving prevalently as hydrolases (lower transglycosylation degree than WT) (see also Supplementary Table S7). In contrast, vivid transglycosylation activity accompanied by the formation of longer chitooligosaccharides was observed in transglycosylation reactions catalyzed by the Ala, Asn, Gln, Gly, and Thr variants. Especially, Glu332Thr *Tf*Hex showed an almost 5-fold higher transglycosylation efficiency compared with WT (Supplementary Table S7). This is a considerable increase in the synthetic potential by introducing a single amino acid exchange.

## 3. Discussion

The present work raises the question of why only a limited number of variants with shifted GalNAcase/GlcNAcase ratio were identified by site-saturation mutagenesis of *Tf*Hex. With 1600 clones screened, over 10-fold the number theoretically required for 99% coverage [42,43], we should have assumed that all six mutations with the significant shift in the activity ratio identified by site-directed mutagenesis would have been detected in the screening after site-saturation mutagenesis. However, this was not the case. Most likely, the ratio and representation of mutant genes in the mixture of plasmids were not completely balanced after site-saturation mutagenesis, and some mutant genes were generated, incorporated into the host chromosome, and expressed preferentially over others. This result indicates that the applicability of Equation (1) by Reetz et al. [42,43] may have practical limitations. Nevertheless, since the overall outcome of this study may lean on the results of both site-directed and site-saturation approaches, we may reasonably suppose that we were able to identify all the mutants at the Glu332 position with a significantly shifted GalNAcase/GlcNAcase ratio.

Another factor that may have contributed to false-positive or false-negative hits in the site-saturation mutagenesis screening is the fact that individual transformants of the same gene in *P. pastoris* may exhibit slightly different catalytic properties (in terms of both specific activity (U/mg) and GalNAcase/GlcNAcase activity ratio); although, with one particular transformant, these parameters are well reproducible in different enzyme productions. Apparently, the activity ratio in β-*N*-acetylhexosaminidases does not solely result from the primary enzyme structure, but other factors must also be considered—besides the optimum folding of the catalytically active tetramer and the glycosylation issue mentioned above, it is also the number of copies of the same gene transformed into the *Pichia* genome and possibly other factors yet undisclosed. From our experience, the catalytic parameters of β-*N*-acetylhexosaminidases produced in *P. pastoris* also slightly vary according to the type of medium and carbon source. Variations between specific activities and yields of different transformants of the same gene (as seen in the Glu332His mutant and also partially in the Glu332Gly mutant prepared by site-directed and site-saturation mutagenesis, cf. Table 1 and Supplementary Table S2) may supposedly be contributed, e.g., to an incomplete folding into an active tetramer comprising two catalytic propeptides. This issue deserves a deeper analysis in the future. The quite heavy workload of screening hundreds of variants further favors the site-directed mutagenesis approach, especially when aiming at single-mutant variants.

In the retrospective analysis of the correlation between site-directed mutagenesis data and in silico predictions, we found that the substrate preference was adequately predicted with the exception of the Glu332Phe variant, which was expected to have higher selectivity based on BioLuminate mutation scan data. However, the values of the calculated differences in the ΔΔ*G* values by mutant scan or ΔXP score for various substrates were poorly correlated with the experimental GlcNAcase/GalNAcase ratio (*R*^2^ lower than 0.1). The lower predictability of the experimental results by molecular modeling reflects the fact that, besides the initial binding affinity, the processing of the substrate also depends on its ability to reach active conformation and proper orientation in the active site. Therefore, not only the binding score (reflecting the substrate affinity) but also the distances to the catalytic residues, the stability of the interactions, and the transition state may play an important role. The main challenge and bottleneck remain in how to weigh the importance of each of these parameters. Moreover, we are still working only with a homology model since *Tf*Hex has never been crystallized, despite many years of effort.

The calculation of energy values for each hydrolysis step, including energy barriers, is possible by the combined QM/MM method. However, structures with a good resolution are required for a precise prediction. Calculation of such parameters for a large number of variants is rather time-consuming. Another complication in predicting some variants is the correct prediction of rotamers [52]; the mutation scan may not fully reflect the enzyme flexibility, especially when the mutation is introduced in the loop region, as in this case. Molecular dynamics simulation helps to sample all possible conformations and optimize the variants, but the scoring problem remains. Despite all these bottlenecks, in this study, the site-directed mutagenesis approach based on in silico modeling provided more information on the relationship between the Glu332 substitution and the GalNAcase/GlcNAcase activity ratio in *Tf*Hex than site-saturation mutagenesis.

## 4. Materials and Methods

### 4.1. General

*p*NP-GlcNAc and *p*NP-GalNAc, standard substrates for β-*N*-acetylhexosaminidases [32], were obtained from Gold Biotechnology, USA. If not stated otherwise, all other chemicals were from VWR Chemicals or Lach-Ner (Czech Republic).

### 4.2. Preparation and Cloning of Mutant Genes

The pPICZαA expression vector carrying the gene of *Tf*Hex WT (GenBank ID: JN601495) and the gene for zeocin resistance were prepared as described earlier [39]. The *Tf*hex gene was cloned downstream of the α-factor-encoding the DNA segment for extracellular protein targeting using *Eco*RI and *Kpn*I restriction sites as described previously [39].

Site-saturation mutagenesis and site-directed mutagenesis were performed using the QuikChange Site-Directed Mutagenesis Kit (Agilent, Santa Clara, CA, USA) and the T-Personal Thermal Cycler (Biometra, Göttingen, Germany) with the following cycling parameters: initial denaturation at 95 °C for 2 min, followed by 18 cycles of denaturation at 95 °C for 20 s, primer annealing for 1 min, elongation at 68 °C for 6 min, and final elongation at 68 °C for 10 min. For each primer pair, the annealing temperature varied, see Supplementary Tables S1 and S3. For site-saturation mutagenesis, four pairs of degenerate primers (Supplementary Table S1) were used to minimize the number of colonies required for screening and also to avoid the GAG codon present in the WT sequence. After amplification, 2 µL of *Dpn*I were added, and the reaction mixture was incubated at 37 °C and 300 rpm for 5 min to cleave the methylated DNA template. The amplified product or product mixture (in the case of site-directed or site-saturation mutagenesis, respectively) was then transformed into *E. coli* Top10 competent cells (Thermo Fisher Scientific, Waltham, MA, USA) by heat shock under the selection pressure of zeocin (100 µg/mL).

In the case of site-directed mutagenesis, the respective mutant plasmid verified by sequencing was amplified, isolated using the High Pure Plasmid Midi Isolation Kit (Roche, Basel, Switzerland), and used for the transformation of *Pichia pastoris* as described further. In the case of site-saturation mutagenesis, all transformants on a plate were pooled and cultivated as a single culture for plasmid isolation. The prepared mixture of mutant plasmids was subsequently used for the transformation of *P. pastoris*.

### 4.3. Transformation of P. pastoris Host with Mutant Genes

The single-mutant pPICZαA-*Tf*Hex plasmid or the mixture of yeast expression vectors pPICZαA carrying various mutant genes (30 µg) were linearized using *Sac*I (New England Biolabs, Ipswich, MA, USA). The *P. pastoris* KM71H cells (Invitrogen, Life Technologies, Waltham, MA, USA) were transformed with the plasmids by electroporation according to the EasySelect *Pichia* Expression Kit standard protocol (Invitrogen, Life Technologies, Waltham, MA, USA). Protein expression data and enzyme activities were determined on a small-scale (site-directed mutants) or in 96-deep-well-plate production systems (saturated mutants).

### 4.4. Deep-Well Screening for Heterologous Expression of β-N-Acetylhexosaminidase Variants in P. pastoris

The 96-deep-well-plate screening cultivation method was used to identify the prospective mutants of *Tf*Hex prepared by site-saturation mutagenesis. The number of colonies needed for the screening was originally calculated according to Reetz et al. [42,43] using Equation (1):

*T* = −*V* ln(1 − *P*_i_) (1)

where *T* is the required number of transformants, *V* denotes the number of required codon combinations (32 in our case), and *P*_i_ is the probability with which the sequence occurs in the library. For our library, the number of transformants was found to be ca. 150 with 99% probability. Finally, 1600 variants were screened to maximize the coverage.

The screening was optimized based on the procedure by Weis and coworkers [44]. Single colonies were inoculated into the wells containing 250 µL of BMD medium (10 g/L glucose, 200 mM K-P buffer, 13.4 g/L yeast nitrogen base, 0.4 mg/L biotin). The cultures were grown under standard conditions (28 °C, 320 rpm, 80% relative humidity) for 60 h. Then, protein expression was induced by adding 250 µL of BMM2 medium (1% methanol, 200 mM K-P buffer, 13.4 g/L yeast nitrogen base, 0.4 mg/L biotin) and incubating under the same conditions for 8 h. The cultures were then fed with 50 µL BMM10 medium (5% methanol, 200 mM K-P buffer, 13.4 g/L yeast nitrogen base, 0.4 mg/L biotin) at time points of 68, 80, and 92 h after inoculation. Then, the cultures were centrifuged and the supernatants were tested for β-*N*-acetylhexosaminidase (GlcNAcase, GalNAcase) activity. Colonies producing enzyme variants with a significantly changed activity were cryopreserved at −80 °C in 15% (*v/v*) glycerol for long-term storage.

### 4.5. Heterologous Expression of β-N-Acetylhexosaminidase Variants in P. pastoris, Screening and Purification

The screening of the expression of site-directed *Tf*Hex mutant variants was performed according to the manufacturer’s instructions (EasySelect *Pichia* Expression Kit; Invitrogen, Life Technologies, USA). For each variant, at least 16 transformants were screened. For the small-scale production of *P. pastoris*, a combination of BMGY medium (Buffered Glycerol Complex Medium) and BMMY medium (Buffered Methanol Complex Medium) was used. Sixteen selected colonies were inoculated into BMGY medium (100 mL) and incubated at 28 °C and 220 rpm overnight. Grown cultures were centrifuged (5000 rpm, 10 min, 12 °C), and the pellets were resuspended in BMMY medium (30 mL), where the recombinant protein expression was induced by methanol (0.5% *v/v*). The cultures were incubated with shaking (28 °C, 220 rpm) for three days, and methanol was added every 24 h. On day five after inoculation, the cultures were tested for the presence of *Tf*Hex using SDS-PAGE and enzyme activity assay (see below). Two colonies for each variant were cryopreserved at −80 °C in 15% (*v/v*) glycerol for long-term storage.

For purification purposes, BMMY medium was exchanged for BMMH (Buffered Methanol Minimal Medium). On day five, the cultures were centrifuged (5000 rpm, 10 min, 12 °C), and the enzyme contained in the supernatant was purified to homogeneity by cation exchange chromatography (Fractogel EMD SO^3−^, Merck, Darmstadt, Germany) using an Äkta Protein Purifier system (Amersham Biosciences, Uppsala, Sweden). Equilibration of the column was performed with 10 mM sodium citrate-phosphate buffer pH 3.5, and the enzyme was eluted with a linear gradient of 0–1 M sodium chloride (60 mL). The purified enzyme was concentrated by ultrafiltration in 50 mM sodium citrate-phosphate buffer pH 5.0, and its purity was verified by SDS-PAGE.

### 4.6. Isolation of Chromosomal DNA

Cryopreserved stocks of *P. pastoris* containing a mutant *Tf*Hex gene (50 µL) were transferred to YPD medium (50 mL, 10 mg/L yeast extract, 20 g/L peptone, 20 mg/L d-glucose), and the culture was incubated overnight (28 °C, 220 rpm). When the cultures reached an *OD*_600_ of 20–30, 4 mL of the culture were centrifuged, and the pellet was resuspended in 50 mM sodium phosphate buffer pH 7.5 containing 10 mM 2-mercaptoethanol. The disruption of the yeast cell wall was promoted by the addition of zymolyase (100 U). Further procedures were performed according to DNeasy UltraClean Microbial Kit (Qiagen; Germany).

### 4.7. Amplification and Ligation of Mutant Genes into the Plasmid for Sequencing

The isolated *Tf*Hex gene was amplified using DreamTaq DNA polymerase (Thermo Fisher Scientific, USA) in a T-Personal Thermal Cycler (Biometra, Germany). The *P. pastoris* chromosomal DNA carrying the *Tf*Hex gene (200 ng) and two pairs of primers were used for PCR amplification. Primer pair 1: Fw: 5’-gctgttttgccattttccaacagcacaaataacggg-3’, Re: 5’-tggtcgacggcgctattcagatcctcttctg-3’; primer pair 2: Fw: 5’-ggggatttcgatgttgctgttttgccattttccaac-3’, Re: 5’-tgatgatggtcgacggcgctattcagatcctc-3’ (Generi Biotech, Hradec Králové, Czech Republic). PCR was run under the following conditions: initial denaturation at 95 °C for 1 min, followed by 35 cycles of denaturation at 95 °C for 30 s, primer annealing at 66 °C for 30 s, elongation at 72 °C for 1 min, and final elongation at 72 °C for 10 min. The PCR product was purified with a GeneJET PCR Purification Kit (Thermo Fisher Scientific, USA). Then, A-overhangs were added to the PCR product using dATP (0.5 mM) and DreamTaq DNA polymerase (Thermo Fisher Scientific, USA). The reaction mixtures were incubated at 70 °C for 15 min and precipitated with sodium acetate and ethanol. The PCR products were ligated into the pGEM-TEasy vector system (Promega, Madison, WI, USA) according to the manufacturer´s protocol and sequenced. At least 5 clones of each variant were confirmed by sequencing to contain the same point mutation. In the case of the combined mutant variant Glu332Trp/Glu332Arg *Tf*Hex (8A11), the ratio of both detected amino acids (in 16 clones) was ca. 3:2.

### 4.8. Protein Characterization

The protein concentration was determined by the Bradford method [53] using Protein Assay Dye Reagent Concentrate (Bio-Rad, Hercules, CA, USA). Calibration was performed for bovine plasma γ-globulin (Bio-Rad, UK).

### 4.9. Enzyme Activity Assay

The hydrolytic activity of β-*N*-acetylhexosaminidases was determined in an end-point assay with spectrophotometric detection. *p*-Nitrophenyl 2-acetamido-2-deoxy-β-d-glucopyranoside (*p*NP-GlcNAc) and *p*-nitrophenyl 2-acetamido-2-deoxy-β-d-galactopyranoside (*p*NP-GalNAc) were used as substrates for determining the GlcNAcase and GalNAcase activity of the enzyme, respectively. The reaction mixture consisted of McIlvaine buffer (50 mM citric acid/100 mM Na_2_HPO_4_, pH 5.0) and 2 mM *p*NP-GlcNAc or *p*NP-GalNAc in a microtube or the well of a microtiter plate. Assay reaction mixtures were pre-incubated at 35 °C, and the reaction was started by adding 10 µL of the appropriately diluted enzyme to avoid exceeding 10% reaction conversion. The reaction ran for 10 min at 850 rpm and was stopped by adding 1 mL of 0.1 M sodium carbonate to the tube or 0.1 mL to the microtiter plate well. The free *p*-nitrophenolate released from the enzymatic reaction was detected by a spectrophotometer at 420 nm. One unit of enzymatic activity is the amount of enzyme that hydrolyzes 1 µmol of the respective substrate per min under the standard assay conditions; all activity determinations were performed at least in triplicate.

Michaelis-Menten parameters of *p*NP-GlcNAc hydrolysis by Glu332Ala, Glu332Asn, Glu332Gln, Glu332Gly, Glu332His, Gly332Lys, or Gly332Thr *Tf*Hex were determined using a discontinuous kinetic assay. The reaction mixture (400 µL) containing *p*NP-GlcNAc, McIlvaine buffer pH 5.0, and the appropriately diluted enzyme was incubated at 25 °C and 850 rpm for 14 min. Aliquots (50 µL) were taken every 2 min, and the reaction was stopped by adding 1 M sodium carbonate (100 µL) to the microplate well. The released *p*-nitrophenolate was determined using a Sunrise Microplate Reader (Tecan, Mennedorf, Switzerland). The kinetic parameters were evaluated by non-linear regression using GraphPad Prism 7 (GraphPad Software, San Diego, CA, USA). In acidophilic glycosidases such as *Tf*Hex, continuous determination of kinetic parameters is not suitable due to a significant overlap of absorption spectra of *p*NP-GlcNAc and *p*-nitrophenol, which results in a poor sensitivity of the measurement.

### 4.10. Analytical Transglycosylation Reactions

*p*NP-GlcNAc (65 mg, 0.19 mmol) and Glu332Ala (1 U, 30.3 µL, 18.7 µg), Glu332Asn (1 U, 0.22 µL, 22.7 µg), Glu332Gln (1 U, 0.33 µL, 21.1 µg), Glu332Gly (1 U, 0.4 µL, 14.6 µg), Glu332His (1 U, 70 μL, 62 µg), Gly332Lys (1 U, 67.6 µL, 3.5 mg), Gly332Thr *Tf*Hex (1 U, 0.3 µL, 2.6 mg), or *Tf*Hex WT (1 U, 0.5 µL, 27 µg) were incubated in 30% acetonitrile/ McIlvaine buffer pH 5 at 35 °C and 1000 rpm. The reaction progress was monitored by TLC (propan-2-ol/water/NH_4_OH *aq*., 7/2/1; visualization by UV and carbonization in 5% H_2_SO_4_ in ethanol) and by HPLC. The reaction was stopped after 5 h by boiling for 2 min. In every analytical reaction, the amount of enzyme was identical (1 U/mL).

HPLC analysis was used to monitor the course of transglycosylation reactions and to quantify the transglycosylation products. The Shimadzu Prominence LC analytical system comprised a Shimadzu CBM-20A system controller, a Shimadzu LC-20AD binary HPLC pump, a Shimadzu CTO-10AS column oven, a Shimadzu SIL-20ACHT cooling autosampler, and a Shimadzu SPD-20MA diode array detector (Shimadzu, Kyoto, Japan). Analyses were performed on a TSK gel Amide-80 column (250 × 4.6 mm, 5 µm) preceded by a TSKgel Amide-80 Guardgel (3.2 × 15 mm, Tosoh corp., Japan) in acetonitrile/water (4/1, *v*/*v*), with gradient elution as follows (A = acetonitrile, B = water): 22% B for 0–7 min, 22–31% B for 7–16 min, 31–22% B for 16–17 min, and 22% B for 17–22 min for column equilibration; flow rate 1 mL/min at 25 °C; injection volume 1 μL: Detection was performed at 200 nm. The calibration curves for respective components of transglycosylation reactions were determined from pure compounds as follows: *p*NP-OH (*A* = 333 797 × *c*, *R*² = 0.9992); *p*NP-GlcNAc (*A* = 463 945 × *c*, *R*² = 1.0000); *p*NP-(GlcNAc)_2_ (*A* = 447 291 × *c*, *R*² = 0.9990); *p*NP-(GlcNAc)_3_ (*A* = 627 451 × *c*, *R*² = 0.9997); *p*NP-(GlcNAc)_4_ (*A* = 480 858 × c, *R*² = 0.9991); *p*NP-(GlcNAc)_5_ (*A* = 480 858 × *c*, *R*² = 0.9991, estimate due to the absence of pure standard); (GlcNAc)_2_ (*A* = 305 237 × *c*, R² = 0.9991); (GlcNAc)_3_ (*A* = 1 560 668 × *c*, *R*² = 0.9991). *A* is the respective peak area in the chromatogram (arbitrary units), and *c* is the compound concentration (mM).

### 4.11. Molecular Modeling, Docking, and Molecular Dynamics

For the modeling and docking of WT and mutant *Tf*Hex, we used the homology model of *Tf*Hex built as described previously [54]. Multiple sequence alignment to determine and compare mutation hotspots was performed by the Mustang algorithm [55] in YASARA [56]. Initial predictions of the effects of mutations were performed by residue scan and mutation in the BioLuminate package of Schrödinger software [49,57]. It uses the MM-GBSA (molecular mechanics with generalized Born and surface area solvation) method [49] for calculating the difference in the free energy of binding for WT and mutant variants. The atoms were parameterized in the OPLS2005 force field and minimized in the implicit VSGB2.0 solvent model.

The models of selected *Tf*Hex variants were then built in YASARA [56] based on the model of WT in complex with docked *p*NP-GlcNAc and *p*NP-GalNAc constructed previously [32]. The best rotamers were selected based on the combination of the SCWALL algorithm [56,58] followed by energy minimization of the mutated residue in vacuum [56] using the YASARA standard protocol.

Modeled structures were placed in the periodic boundary conditions (the box border is extended by 0.1 nm from the system in each direction), and explicit TIP3P water with neutralizing Na^+^ ions was added in YASARA. For the simulation of substrates, we used the GLYCAM force field [59]. The parameters for substrates were assigned from the GLYCAM force field [59]; the parameters for the *p*NP group were automatically added using the AutoSMILE approach [60]; the parameters for the protein, water, and ions were from YASARA2 force field [61]. The protonation state of the residues was assigned for pH 6.0, and the catalytic Glu371 was modeled in the protonated state. The modeled complexes were minimized in YASARA according to the standard protocol and used for further molecular dynamics simulation in the NPT (constant number of particles N, pressure P, and temperature T, adjusted by Berendsen thermostat) ensemble [62]. The results of the molecular dynamics simulation were analyzed by YASARA tools [63] and visualized by Xmgrace [64].

The equilibrated systems for calculating the Glide XP binding score were selected from molecular dynamics simulations based on the stable RMSD values and a short distance from the catalytic Glu371 residue, as explained previously [32].

## 5. Conclusions

By employing site-directed mutagenesis based on rational design, we performed a detailed study on the influence of a point mutation of the Glu332 residue in the active site of *Tf*Hex, a promiscuous enzyme, on its substrate specificity. Two variants, Glu332His and Glu332Lys *Tf*Hex, were the most selective GlcNAcases with an 8-fold and 7-fold higher activity, respectively, towards *p*NP-GlcNAc over *p*NP-GalNAc compared with WT. Their synthetic potential was tested in a transglycosylation reaction to determine that they both behaved more like hydrolases, forming fewer transglycosylation products than WT. This property may be used for selective cleavage of GlcNAc-terminated oligosaccharides from reaction mixtures. Five other variants containing Ala, Asn, Gln, Gly, or Thr at position 332 combined a higher specific GlcNAcase activity than WT with a high transglycosylation capability, producing almost five times more transglycosylation products than WT.

Based on this and previous work [32], we conclude that the task of shifting the GalNAcase/GlcNAcase activity ratio in transglycosylating β-*N*-acetylhexosaminidases is extremely challenging and that it might even be generally beyond reach to acquire a very high substrate selectivity (>100-fold) in these enzymes (as also apparent in the existing WT enzymes of this class [32]. In this study, site-saturation mutagenesis followed by high-throughput screening gave less information on the structure-activity relationship than rational site-directed mutagenesis. This is also caused by the strong dependence of catalytic properties of β-*N*-acetylhexosaminidases on the enzyme folding, glycosylation pattern, cultivation medium, and higher complexity of the expression system of *P. pastoris*, which is, however, necessary for correct folding of propeptide-carrying tetramers of fungal β-*N*-acetylhexosaminidases such as *Tf*Hex.

## Figures and Tables

**Table 1:** Purification yields and specific activities of site-directed mutants of *Tf*Hex.

Glu332 Substitution	Yield ^a^ [mg]	GlcNAcase [U/mg]	GalNAcase [U/mg]	GalNAcase/ GlcNAcase
WT ^b^	5.1 ± 0.2	35 ± 2	42 ± 2	1.2
Ala	0.33 ± 0.05	54 ± 1	12.9 ± 0.5	0.24
Asn	15.5 ± 0.6	44 ± 2	17.1 ± 0.3	0.39
Gln	9.5 ± 0.3	47 ± 1	13.6 ± 0.4	0.29
Gly	18.3 ± 0.7	68 ± 2	17.7 ± 0.6	0.26
His	5.7 ± 0.2	8.8 ± 0.1	1.32 ± 0.02	0.15
Lys	9.9 ± 0.2	1.14 ± 0.04	0.21 ± 0.05	0.18
Phe	10.6 ± 0.4	7.3 ± 0.1	7.4 ± 0.2	1.0
Thr	13.1 ± 0.3	138 ± 3	30 ± 1	0.22
Trp	13.7 ± 0.3	10.5 ± 0.3	4.9 ± 0.1	0.47
Tyr	8.8 ± 0.1	4.75 ± 0.04	6.65 ± 0.05	1.4

^a^ Purification from 30 mL media. ^b^ Values are in accord with the literature [23].

## Data Availability

Data can be obtained from the corresponding author upon reasonable request.

## Supplementary Materials

The supporting information can be downloaded at: https://www.mdpi.com/article/10.3390/ijms232012456/s1.
