# Supplementary material for: Mutation Hotspot for Changing the Substrate Specificity of β-N-Acetylhexosaminidase: A Library of GlcNAcases
Source: Int J Mol Sci. 2022 Oct 18;23(20):12456. doi: 10.3390/ijms232012456 (PMC9604439; doi:10.3390/ijms232012456)
Supplement: Supplementary file 1 [file ijms-23-12456-s001.zip › ijms-1914550-supplementary.pdf]

International Journal of Molecular Sciences

Supplementary Materials

Mutation Hotspot for Changing the Substrate specificity of β-*N*-Acetylhexosaminidase: A Library of GlcNAcases

Pavlína Nekvasilová [1,2], Natalia Kulik [3], Michael Kotik [1], Lucie Petrásková [1], Kristýna Slámová [1], Vladimír Křen [1], and Pavla Bojarová [1,*]

1 Laboratory of Biotransformation, Institute of Microbiology of the Czech Academy of Sciences, Vídeňská 1083, Praha 4, CZ-14220, Czech Republic.
2 Department of Genetics and Microbiology, Faculty of Science, Charles University, Viničná 5, Praha 2, CZ-12843, Czech Republic.
3 Laboratory of Structural Biology and Bioinformatics, Institute of Microbiology of the Czech Academy of Sciences, Zámek 136, Nové Hrady, CZ-37333, Czech Republic.

* Corresponding author: Pavla Bojarová. E-mail: bojarova@biomed.cas.cz

Contents

1. Site-saturation Mutagenesis
2. Site-directed Mutagenesis
3. Molecular Modeling
4. Analysis by SDS-PAGE and Catalytic Properties of Mutant *Tf*Hex Variants

Site-saturation Mutagenesis

Table S1. Primers used for site-saturation mutagenesis of *Tf*Hex[a].

Code	Primer sequence[b]	Annealing temperature
Pair 1	Fw: 5'-tggccttaccatactgccgtt**yrn**cctacccccggtc-3'<br>Re: 5'-gaccgggggtagg**nyr**aacggcagtatggtaaggcca-3'	68-64.4 °C, Δ -0.2[c]
Pair 2	Fw: 5'-tggccttaccatactgccgtt**ans**cctacccccggtc-3'<br>5'-gaccgggggtagg**snt**aacggcagtatggtaaggcca-3'	65-61.4 °C, Δ -0.2[c]
Pair 3	Fw: 5'-gccttaccatactgccgtt**gnt**cctacccccggt-3'<br>Re: 5'-accgggggtagg**anc**aacggcagtatggtaaggc-3'	62-58.4 °C, Δ -0.2[c]
Pair 4	Fw: 5'-tggccttaccatactgccgtt**yyt**cctacccccggtc-3'<br>Re: 5'-gaccgggggtagg**arr**aacggcagtatggtaaggcca-3'	62-58.4 °C, Δ -0.2[c]

[a] To verify the efficiency of site-saturation mutagenesis, the mixture of the pPICZαA-*Tf*Hex mutant clones was transformed into *E. coli* Top10 cells, and sample plasmids were isolated and sequenced. Over 90% of the isolated genes carried a point mutation at the position 332. The sequencing also revealed several site-directed mutant variants that were later examined in the site-directed mutagenesis part of this work.
[b] Four pairs of degenerate primers were used to minimize the number of colonies required for screening, and to cover all 19 variants, avoiding the GAG codon present in the wild-type sequence.
[c] Touch-down PCR.

Table S2. Purification yield, specific activities, and identified mutation at the position 332.

Code	Yield [mg]	GlcNAcase [U/mg]	GalNAcase [U/mg]	*GalNAcase/ GlcNAcase ratio*	Identified AA
WT[a]	5.1 ± 0.2	35 ± 2	42 ± 2	*1.2*	Glu
3G8	3.8 ± 0.1	0.62 ± 0.02	0.11 ± 0.01	*0.17*	His
8A11	6.4 ± 0.2	6.0 ± 0.3	2.1 ± 0.2	*0.34*	Trp, Arg
8H11	17.0 ± 0.7	4.4 ± 0.5	1.9 ± 0.5	*0.42*	Trp
11H6	13.8 ± 0.6	39.2 ± 0.9	11.49 ± 0.07	*0.29*	Gly
12D10	4.3 ± 0.1	53 ± 1	11.6 ± 0.4	*0.22*	Gly
14E7	4.9 ± 0.3	46.9 ± 0.8	14.2 ± 0.3	*0.30*	n.d.[b]
14G12	2.7 ± 0.3	57 ± 2	13.6 ± 0.4	*0.24*	n.d.[b]

[a] Values are in accord with the literature [1].
[b] n.d., not determined

Site-directed Mutagenesis

Table S3. PCR primers used for site-directed mutagenesis of *Tf*Hex.

Mutant	Template	Primer sequence	Annealing temperature
**Glu332Ala**	Glu332Gly	Fw: 5'-catactgctcctacccccggt-3'<br>Re: 5'-accgggggtaggagcaacggcagtatg-3'	61 °C
**Glu332Gln**	WT	Fw: 5'-ccttaccatactgccgttcagcctacccc-3'<br>Re: 5'-ggggtaggctgaacggcagtatggtaagg-3'	61 °C
**Glu332His**	Glu332Tyr	Fw: 5'-ccttaccatactgccgttcatcctacccccgg-3'<br>Re: 5'-ccgggggtaggatgaacggcagtatggtaagg-3'	63 °C
**Glu332Trp**[a]<br>**Glu332Tyr**[a]	WT	Fw: 5'-tggccttaccatactgccgtt**yrn**cctacccccggtc-3'<br>Re: 5'-gaccgggggtagg**nyr**aacggcagtatggtaaggcca-3'	68-64.4 °C, Δ -0.2[b]
**Glu332Asn**[a]<br>**Glu332Lys**[a]<br>**Glu332Thr**[a]	WT	Fw: 5'-tggccttaccatactgccgtt**ans**cctacccccggtc-3'<br>Re: 5'-gaccgggggtagg**snt**aacggcagtatggtaaggcca-3'	65-61.4 °C, Δ -0.2[b]
**Glu332Gly**[a]	WT	Fw: 5'-gccttaccatactgccgtt**gnt**cctacccccggt-3'<br>Re: 5'-accgggggtagg**anc**aacggcagtatggtaaggc-3'	62-58.4 °C, Δ -0.2[b]
**Glu332Phe**[a]	WT	Fw: 5'-tggccttaccatactgccgtt**yyt**cctacccccggtc-3'<br>Re: 5'-gaccgggggtagg**arr**aacggcagtatggtaaggcca-3'	62-58.4 °C, Δ -0.2[b]

[a] Mutant variants were obtained in the frame of site-saturation mutagenesis.

[b] Touch-down PCR

Table S4. Codons present in *Tf*Hex variants.

Substitution of Glu332	Codon
**WT**	gag
**Ala**	gct
**Asn**	aac
**Gln**	cag
**Gly**	ggt
**His**	cat
**Lys**	aag
**Phe**	ttt
**Thr**	acc
**Trp**	tgg
**Tyr**	tat

Molecular Modeling

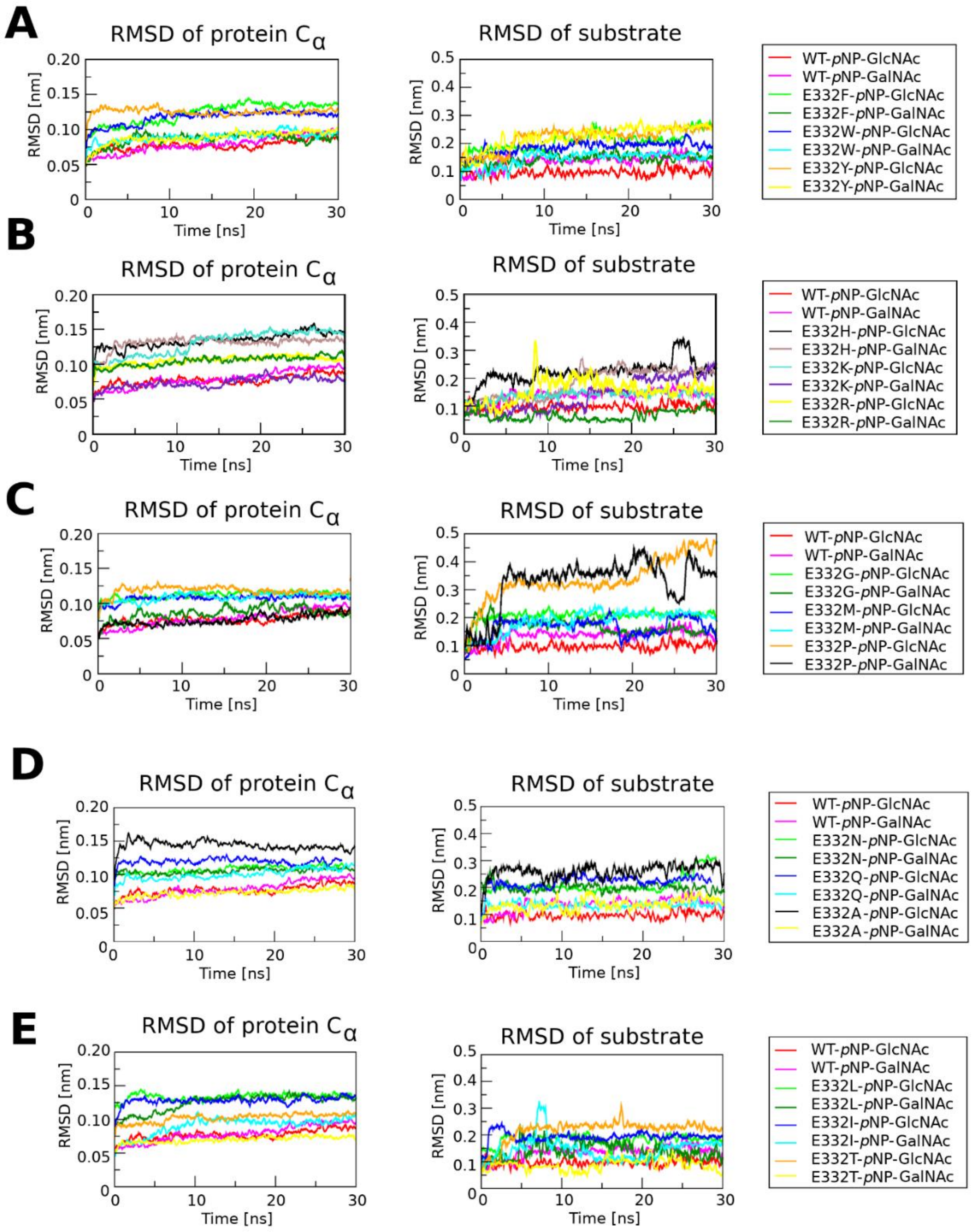

Figure S1. RMSD (root-mean-square deviation) of C-α atoms of the enzyme (left panel) and substrate atoms (right panel) during molecular dynamics simulation. Data are averaged for 10 snapshots. The highest root-mean-square deviation (RMSD) of substrates with respect to the initial docked position was found for Glu332Pro *Tf*Hex, especially for *p*NP-GlcNAc (Figure S1C). This indicates a low stability of this enzyme-substrate complex. *p*NP-GlcNAc was not stabilized (fluctuations in RMSD) in the active site of Glu332Met, and Glu332Arg *Tf*Hex; *p*NP-GalNAc was rather flexible in the active site of Glu332His, Glu332Lys, and Glu332Gly *Tf*Hex. Neither of the substrates was well stabilized in the active site of Glu332Leu and Glu332Ile *Tf*Hex (Figure S1E).

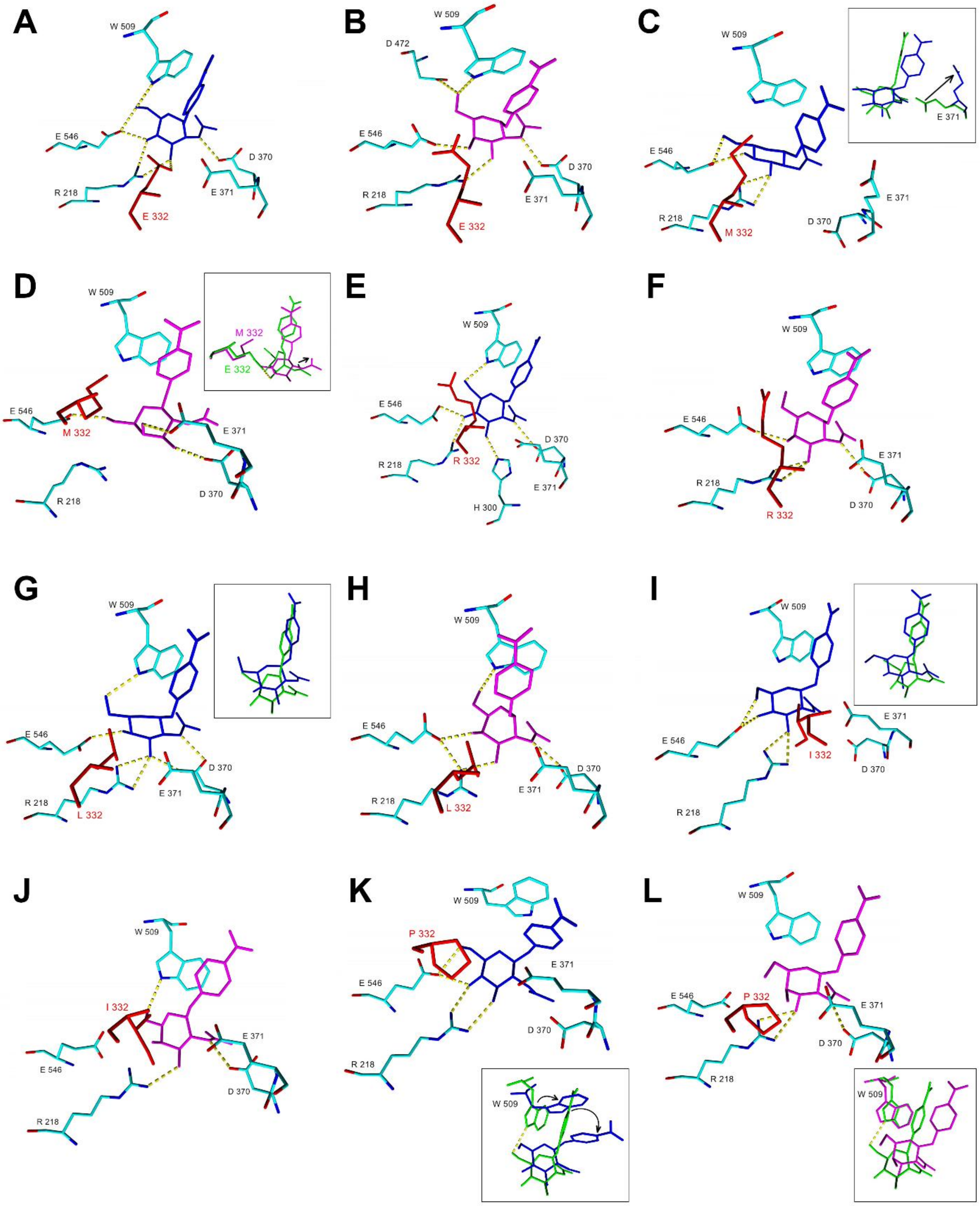

Figure S2. Orientation of substrates in equilibrated substrate-enzyme complexes. *p*NP-GlcNAc is in **blue**, *p*NP-GalNAc in **magenta**, and the residue at the position 332 is in **red**. The black arrow shows the main changes. **A**. WT–*p*NP-GlcNAc; **B**. WT–*p*NP-GalNAc; **C**. Glu332Met–*p*NP-GlcNAc. The inset represents a comparison of the position of the substrate and catalytic Glu371 in the Glu332Met mutant (**blue**) and WT (**green**); **D**. Glu332Met–*p*NP-GalNAc. The inset represents a comparison of substrate position in the Glu332Met mutant (**magenta**) and WT (**green**); **E**. Glu332Arg–*p*NPGlcNAc; **F**. Glu332Arg–*p*NP-GalNAc; **G**. Glu332Leu–*p*NP-GlcNAc. The inset represents a comparison of the substrate position in the Glu332Leu mutant (**blue**) and WT (**green**); **H**. Glu332Leu–*p*NP-GalNAc; **I**. Glu332Ile–*p*NP-GlcNAc. The inset represents a comparison of the substrate position in the Glu332Ile mutant (**blue**) and WT (**green**); **J**. Glu332Ile–*p*NP-GalNAc; **K**. Glu332Pro–*p*NP-GlcNAc. The inset represents a comparison of the substrate position in the Glu332Pro mutant (**blue**) and

WT (**green**); **L**. Glu332Pro–*p*NP-GalNAc. The inset represents a comparison of the substrate position in the Glu332Pro mutant (**magenta**) and WT (**green**). Extensive fluctuations in substrate orientation, substrate distortions, and excessive distances between the glycosidic bond and the Glu332 residue were observed particularly for Glu332Met *Tf*Hex (Figures S2C,D), and for the mutants with an exchange for aliphatic amino acid residues (Glu332Leu and Glu332Ile *Tf*Hex; Figures S2G-J). In Glu332Pro *Tf*Hex, both substrates were shifted closer to Trp509 and lost hydrogen bonding with Asp472 and Trp509; *p*NP-GalNAc also rotated and lost hydrogen bonding with Glu546 (Figure S2K-L). In the *p*NP-GlcNAc-Glu332Pro complex, Trp509 flipped with a different final orientation (Figure S2K).

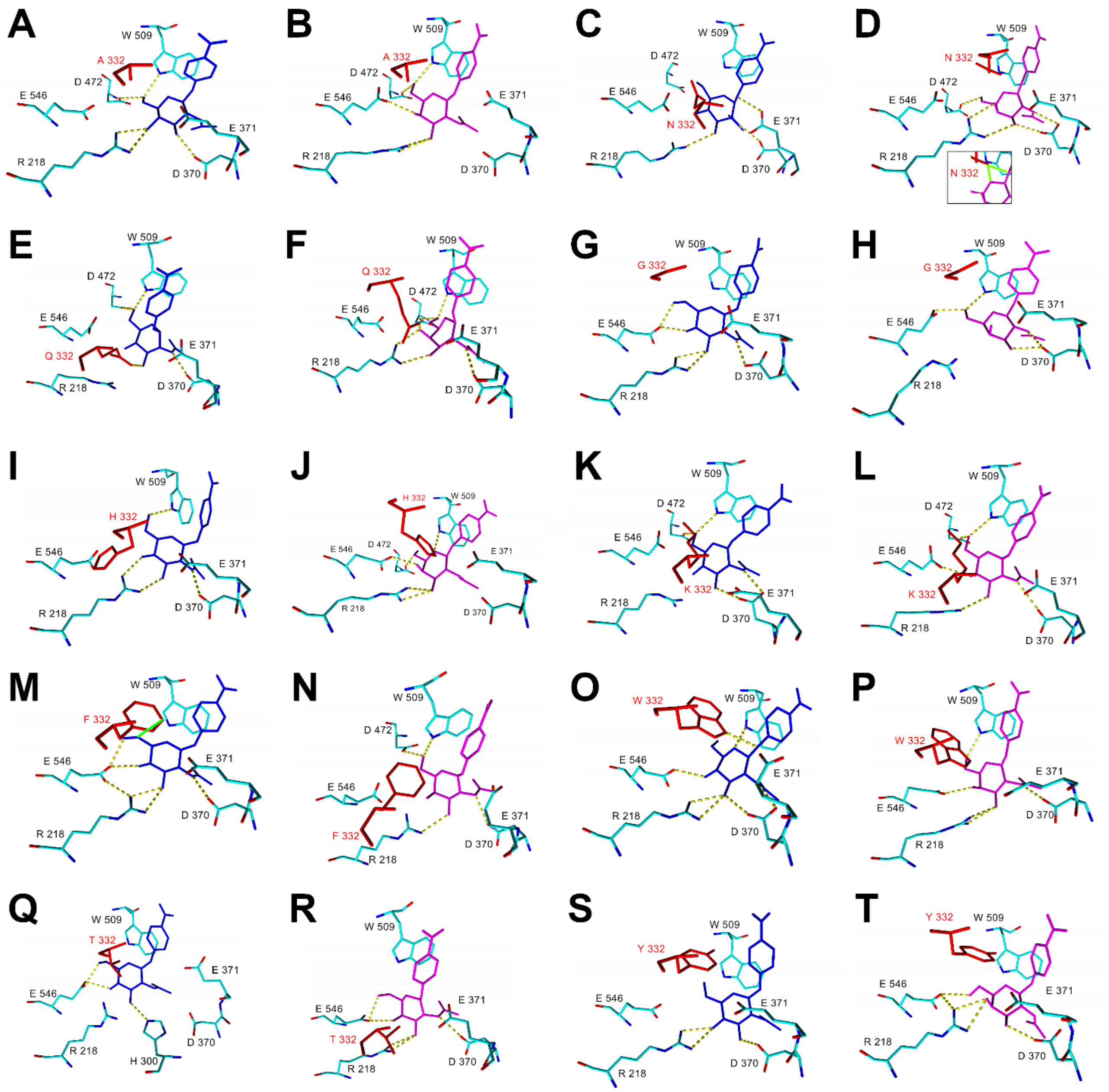

Figure S3. Orientation of substrates in the equilibrated substrate-enzyme complexes. *p*NP-GlcNAc is in **blue**, *p*NP-GalNAc in **magenta**, the residue at position 332 in **red**: **A**. Glu332Ala–*p*NP-GlcNAc: *p*NP-GlcNAc orientation changed during molecular dynamics – it shifted deeper in the active site than in WT, and Asp370 formed new interactions with its C-3 hydroxyl; **B**. Glu332Ala–*p*NP-GalNAc: *p*NP-GalNAc orientation was similar to WT and it rarely formed hydrogen bonding with the catalytic nucleophile Asp370 (see also Figure S4B); **C.** Glu332Asn–*p*NP-GlcNAc; **D**. Glu332Asn–*p*NP-GalNAc: Asn332 formed an unusual hydrogen bond with the pyranose oxygen or the glycosidic oxygen of *p*NP-GalNAc (**green** lines in inset) while this bond could not be formed in *p*NP-GlcNAc due to the equatorial C-4 hydroxyl; **E.** Glu332Gln–*p*NP-GlcNAc: mutation

caused changes in hydrogen bonding in the active site - *p*NP-GlcNAc lost hydrogen bond with Arg218, rarely formed hydrogen bond with Glu546, and Gln332 newly formed hydrogen bond with Arg218 or Glu546. The C-3-C-5 atoms of *p*NP-GlcNAc shifted further from their initial position (see also Figure S1D, **blue** line), which led to a larger distance from catalytic Glu371 after 20 ns of molecular dynamics; **F**. Glu332Gln–*p*NP-GalNAc: during molecular dynamics, Gln332 formed hydrogen bonding not only with C-3 and/or C-4 hydroxyl of *p*NP-GalNAc but also with the pyranose ring oxygen (similar to Asn332 in the Glu332Asn–*p*NP-GalNAc complex), which hampered transition state formation, interaction with catalytic Asp370 was unstable (see also Figure S4); **G.** Glu332Gly–*p*NP-GlcNAc: mutation caused enlargement of the active site close to Gly332, and hence better water accessibility; **H**. Glu332Gly–*p*NP-GalNAc: the average distance between the catalytic Glu371 and glycosidic oxygen of pNP-GalNAc increased, which may slow down product formation; **I.** Glu332His–*p*NP-GlcNAc: *p*NP-GlcNAc lost hydrogen bond with Asp472 and Glu332, and its pyranosyl ring was rotated in the active site (see also Figure S1B, **black** line); **J**. Glu332His–*p*NP-GalNAc: pNP-GalNAc fomed comparable number of hydrogen bonds with WT but the unusual hydrogen bond between Trp509 and pyranosyl oxygen of *p*NP-GalNAc may influence proper formation of the transition state; **K.** Glu332Lys–*p*NP-GlcNAc; **L**. Glu332Lys–*p*NP-GalNAc: Asp370 formed a hydrogen bond with *p*NP-GalNAc more rarely than with *p*NP-GlcNAc (see also Figure S4B); **M.** Glu332Phe–*p*NP-GlcNAc: *p*NP-GlcNAc was rotated in the active site (see also Figure S1A, **light green** line), and lost interaction with Asp472 and Trp509 during molecular dynamics while it formed stronger interaction with Glu546 and Arg218 and hydrophobic interaction with Phe332; **N**. Glu332Phe–*p*NP-GalNAc: hydrophobic interaction between *p*NP-GalNAc and Phe332 was not formed and the distance to catalytic Glu371 was slightly higher than with *p*NP-GlcNAc but the substrate position was well preserved; **O.** Glu332Trp–*p*NP-GlcNAc: Trp509 may form a hydrogen bond with pyranosyl oxygen of *p*NP-GlcNAc, which may cause problems during transition state formation; otherwise interaction similar to WT; **P**. Glu332Trp–*p*NP-GalNAc: interactions similar to WT; **Q.** Glu332Thr–*p*NP-GlcNAc; **R**. Glu332Thr–*p*NP-GalNAc; **S.** Glu332Tyr–*p*NP-GlcNAc: orientation of Asp370 changed significantly; **T**. Glu332Tyr–*p*NP-GalNAc: catalytic Glu371 was distorted similar to *p*NP-GlcNAc and rotated closer to C-4 of *p*NP-GalNAc but Asp370 orientation was closer to WT, and the number of hydrogen bonds was higher than in the Glu332Tyr–*p*NP-GlcNAc complex.

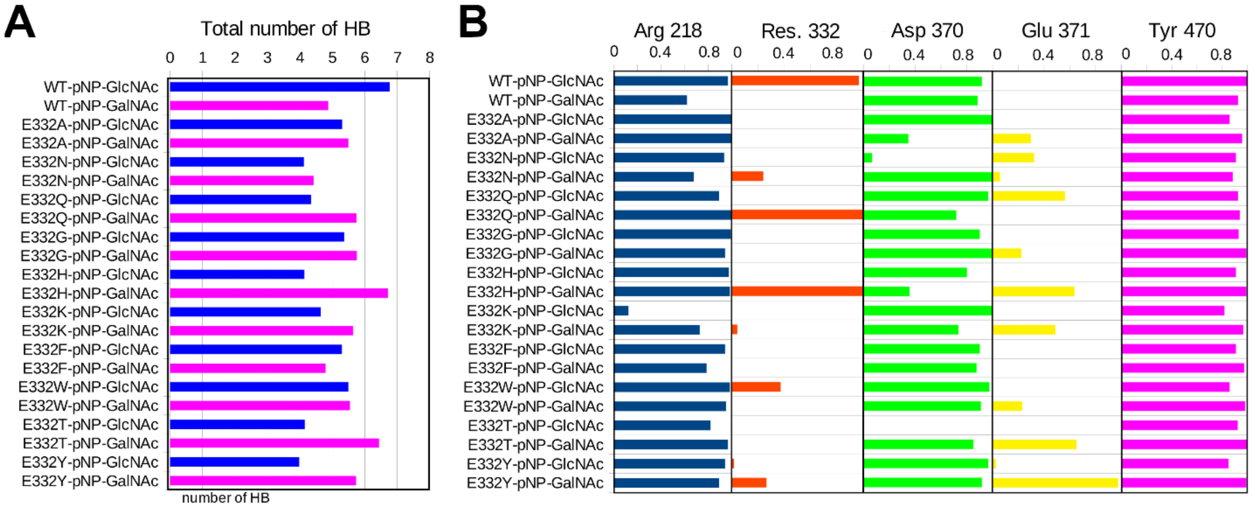

Figure S4 A. The total number of hydrogen bonds formed by substrates during the stable period of molecular dynamics simulation; **B.** the frequency of formation of hydrogen bonds with selected active site residues during molecular dynamics simulation. Frequency was calculated as a ratio of the number of snapshots with hydrogen bonds and the total number of snapshots.

Table S5. Binding XP scores for *Tf*Hex variants with *p*NP-GlcNAc and *p*NP-GalNAc substrates.

Enzyme	Binding XP score [kJ/mol]		Difference in XP score[a] [kJ/mol]
	*p*NP-GlcNAc	*p*NP-GalNAc	
WT	-38.5[b]	-34.9[b]	3.6
**Glu332Phe**	**-29.2**	**-35.5**	**6.3**
**Glu332Gly**	**-27.9**	**-32.6**	**4.7**
**Glu332His**	**-25.0**	**-30.5**	**5.6**
Glu332Lys	-32.8	-36.0	3.2
**Glu332Asn**	**-24.2**	**-28.2**	**4.0**
Glu332Gln	-39.3	-37.6	2.7
Glu332Ala	-33.9	-31.5	2.4
Glu332Trp	-34.6	-32.5	2.1
Glu332Thr	-30.1	-31.6	1.5
Glu332Tyr	-27.3	-29.9	2.6

[a] Difference in XP score was calculated as the difference between the XP score of *p*NP-GlcNAc and the XP score of *p*NP-GalNAc. For the sake of simplifying the message, an absolute value is considered.
[b] Data were adopted from the literature [1].

Analysis by SDS-PAGE and Catalytic Properties of Mutant *Tf*Hex Variants

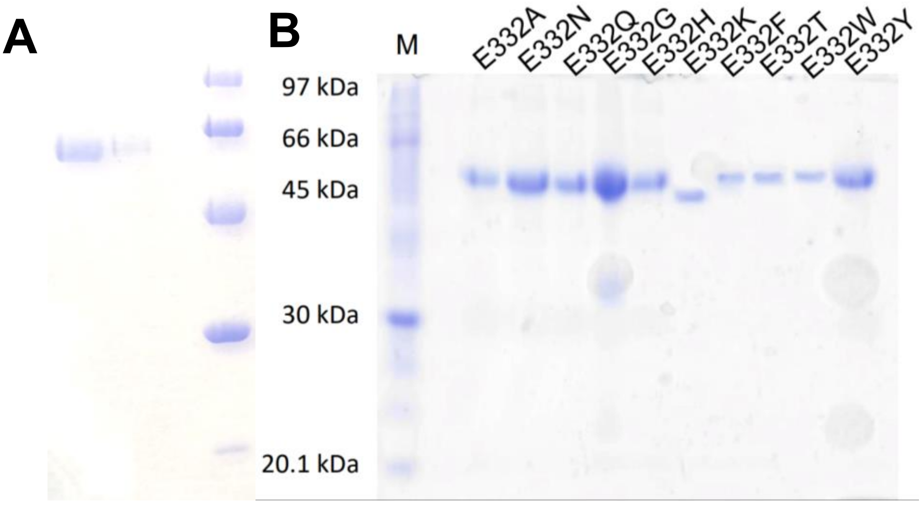

Figure S5. A. SDS-PAGE of WT *Tf*Hex (left: *Tf*Hex WT; right: molecular mass marker), **B.** SDS-PAGE of *Tf*Hex variants after purification by cation exchange chromatography. Lane M, molecular mass marker: phosphorylase b from rabbit muscle (97 kDa), bovine serum albumin (66 kDa), chicken egg-white ovalbumin (45 kDa), carbonic anhydrase from bovine erythrocytes (30 kDa), and trypsin inhibitor from soya beans (20.1 kDa). Different MWs of enzymes indicate different degrees of glycosylation by the *Pichia* host as also shown previously [3].

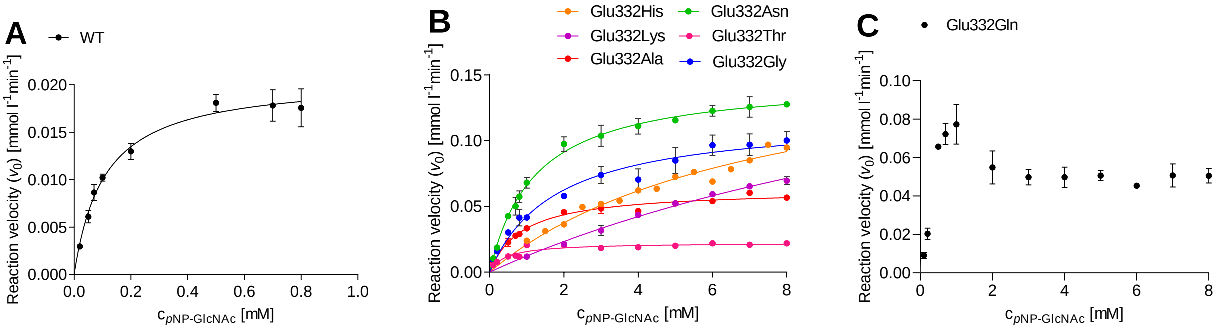

Figure S6 A. Michaelis-Menten kinetic curve of hydrolysis of *p*NP-GlcNAc (0.01 – 0.8 mM) by *Tf*Hex WT. **B.** Michaelis-Menten kinetic curve of hydrolysis of *p*NP-GlcNAc **(**1.0 – 8.0 mM) by selected *Tf*Hex variants. The kinetic parameters (K_M, k_{cat}) were obtained from non-linear regression using GraphPad Prism 7 (GraphPad Software, USA). For Glu332His *Tf*Hex, the values are just estimates due to the low saturation of the kinetic curve caused by limited substrate solubility in higher concentrations. **C.** The dependence of the rate of hydrolysis of *p*NP-GlcNAc (1.0 – 8.0 mM) on its concentration catalyzed by Glu332Gln *Tf*Hex. The course of the curve did not fit any Michaelis-Menten kinetic model, which might be caused by changes in hydrogen bond interactions between active-site residues. During molecular dynamics simulation, Arg218 or Glu546 formed hydrogen bonds with Gln332 instead of *p*NP-GlcNAc (see also Figure S3E). The changes in substrate position caused an increase in the distance of *p*NP-GlcNAc from the catalytic residue Glu371 after 20 ns of molecular dynamics simulation. Due to the imprecise fit of the kinetic curve, we do not consider the extraction of kinetic parameters justified for Glu332Gln *Tf*Hex (denoted as n.d. in Table S6).

Table S6. Kinetic parameters of *p*NP-GlcNAc hydrolysis by selected *Tf*Hex variants and comparison with literature.

**Enzyme**	**GlcNAcase [U/mg]**	K_M **[mM]**	k_{cat} **[s^{-1}]**	k_{cat}/K_M **[s^{-1} mM^{-1}]**
***Tf*Hex WT**	37	0.11 ± 0.02	47 ± 1	434
***Av*Hex WT**[a]	46	0.18 ± 0.03	8.1 ± 0.3	45
**Arg218Lys *Tf*Hex**[b]	36	0.9 ± 0.2	29 ± 1	32
**Glu332Ala *Tf*Hex**	54	0.88 ± 0.07	30 ± 1	34
**Glu332Asn *Tf*Hex**	44	1.22 ± 0.07	22.5 ± 0.4	18
**Glu332Gln *Tf*Hex**[c]	47	n.d.	n.d.	n.d.
**Glu332Gly *Tf*Hex**	68	1.8 ± 0.2	51 ± 2	28
**Glu332His *Tf*Hex**	8.8	7.4 ± 0.2	1.7 ± 0.1	0.2
**Glu332Lys *Tf*Hex**	1.1	≈ 16	≈ 22.5	1.4
**Glu332Thr *Tf*Hex**	138	0.8 ± 0.1	13.3 ± 0.4	16

[a] Values were adopted from the literature [2].

[b] Values were adopted from the literature [3].

[c] Kinetic parameters were not determined for the Glu332Gln variant due to the imprecise fit to the Michaelis-Menten curve, caused probably by the loss of interaction of the enzyme catalytic residues with *p*NP-GlcNAc.

n.d., not determined

Table S7. Contents of reactants and products in the transglycosylation reaction with WT and mutant *Tf*Hex variants (see Figure 3 in the main text) after 5 h.

Compound[a]	Concentration ratio of reaction components [%][b]							
	WT	Glu332Ala	Glu332Asn	Glu332Gln	Glu332Gly	Glu332His	Glu332Lys	Glu332Thr
*p*NP-OH	45.0	30.0	25.7	40.4	31.5	7.8	22.7	37.6
*p*NP-GlcNAc	29.9	43.3	48.9	24.4	38.4	91.8	54.1	28.0
GlcNAc	19.8	7.3	8.0	17.5	5.5	0.1	21.0	7.4
*p*NP-(GlcNAc)$_2$	2.4	17.0	13.9	10.5	17.9	0.4	1.5	17.4
*p*NP-(GlcNAc)$_3$	0.3	0.7	2.5	2.9	4.6			5.2
*p*NP-(GlcNAc)$_4$		0.9	0.4	1.0	1.6			2.7
*p*NP-(GlcNAc)$_5$	0.1	0.1	0.1	0.7	0.3		0.4	0.9
(GlcNAc)$_2$	2.6	0.6	0.4	2.5	0.3		0.4	0.8
(GlcNAc)$_3$	0.3			1.0				0.7
**sum of transglycosylation products**	**5.6**	**19.3**	**17.4**	**18.6**	**24.7**	**0.4**	**2.2**	**27.7**

[a] Substrate is in **red**, hydrolytic product in **blue**, and transglycosylation products in **green.**

[b] Determined by HPLC analysis; for conditions see the main text.

References

1. Bojarová, P.; Kulik, N.; Hovorková, M.; Slámová, K.; Pelantová, H.; Křen, V., The β-*N*-acetylhexosaminidase in the synthesis of bioactive glycans: Protein and reaction engineering. *Molecules* **2019,** 24, 599.
2. Bojarová, P.; Kulik, N.; Slámová, K.; Hubálek, M.; Kotik, M.; Cvačka, J.; Pelantová, H.; Křen, V., Selective β-*N*-acetylhexosaminidase from *Aspergillus versicolor* - a tool for producing bioactive carbohydrates. *Appl. Microbiol. Biotechnol.* **2019,** 103, 1737-1753.
3. Nekvasilová, P.; Kulik, N.; Rychlá, N.; Pelantová, H.; Petrásková, L.; Bosáková, Z.; Cvačka, J.; Slámová, K.; Křen, V.; Bojarová, P., How site-directed mutagenesis boosted selectivity of a promiscuous enzyme. *Adv. Synt. Catal.* **2020,** 362, 4138-4150.
